# Practical Observations on Acute and Chronic Carditis

**Published:** 1818-06

**Authors:** M. Merat


### *Practical Observations on Acute and Chronic Carditis.* By M. Merat.

It is extremely difficult, perhaps impossible, to distinguish carditis from pericarditis; and from the immediate vicinity of other organs, often simultaneously affected with inflammation, it is not always that we can say positively that pericarditis *alone* is present, except in very *acute* cases, which are comparatively rare. Most frequently carditis is slow, obscure, insidious, and, as it were, masked; and it is only by dissection that we learn the true nature of the disease. Corvisart regards carditis as affecting, at the same time, the various tissues of the heart; that is, the muscular, serous, and vascular structures. That the disease is seldom *acute*, may be inferred from the experience of Corvisart himself, who declares that he never saw a single instance of it, and only admits of its existence from the observations of others.

1. *Etiology*. The causes of carditis are those of inflammation in other viscera. Alternations of heat and cold, violent exercise beyond the strength, suppressed perspiration, suspension of natural evacuations, rheumatic metastasis, &c. are the principal agents in the production of carditis. It is in general an extension of pulmonic or pleuritic inflammation from the contiguous parts to the organ of the circulation.

2. *Symptomatology*. Selle characterizes carditis by the following phænomena:—Pungent pain under the sternum; palpitation of the heart; constant anxiety; small, unequal pulse; heat not excessive. Stoll and Pinel add, a sense of burning in the præcordial region; an obtuse pain, and a constriction round the heart, with anxiety, inquietude, syncope, feeble pulse, &c. In the *Journal de Medicine* for 1755, we find, an extreme difficulty of breathing, with a long interval between expiration and inspiration. I have myself remarked, a sense of burning heat in the præcordia, but the pulse has seldom appeared feeble and soft; on the contrary, it was generally frequent and full, as in other active inflammations.

3. *March and Duration*. The symptoms of acute carditis progressively increase in violence. The pulse becomes more concentrated and unequal; the pain and anxiety increase as the disease approaches to a fatal termination, which generally takes place. The prognosis is unfavourable. Probably the fatality of this disorder is owing to the circumstance of the heart being in constant action while in a state of inflammation, which must greatly aggravate the complaint. The sufferings of a patient labouring under carditis, may be guessed at by those in a muscle, when exercised during rheumatic inflammation. If recovery takes place, the symptoms become gradually milder; the pulse more natural and regular; the anxiety and oppression diminish; and finally the patient emerges into convalescence. The chronic or occult species has no fixed limits as to duration—it may go on for months; nay, for years.

4. *Pathology.* Dissection is more satisfactory than the symptomatology. We generally find in carditis, the muscular fibres of the heart converted into a soft and pale substance, of little tenacity. Sometimes they are penetrated and separated by a purulo-lymphatic fluid. The vascular system of the organ is more apparent than usual, and partakes in the inflammatory state of the other tissues. When the pericardium is stripped off, there often appears a layer of pale and yellow greasy substance on the surface of the heart, the fleshy parietes of which are easily lacerable, and seem like bouillie. In the cavities, a mass of coagula and gelatinous concretions are found, owing to the stagnation of blood in the last hours of life, the heart being incapable of propelling it through the vascular system. Instead of these appearances, we sometimes find a large quantity of purulent matter in the cavity of the pericardium, or ulcerations on the surface of the organ itself.

5. *Treatment.* General and local bleeding; saline purgatives; *absolute repose;* diluents. To these remedies of the French physician, we would add antimonials, digitalis, and the most powerful counter-irritants, both on the thorax and the extremities. But above all, in *chronic carditis,* which is the form most usually met with, we would strongly recommend the introduction of mercury, so as to saturate the system, a remedy which we have strong reason to believe has saved the lives of several patients under our own care, who laboured under this insidious disease.

*Case from the French.*

A woman, 36 years of age, who had suffered three severe attacks of *acute rheumatism,* was seized in the night of the 5th of November, 1809, with general horripilation, tumultuous movements of the heart, a lancinating pain in the præcordial region, difficult respiration, lypothymia of an hour's duration. She had neither thirst nor cough. Antispasmodics and rubefacients to the lower extremities produced no change in the symptoms.

On the 4th day, the patient was received into the hospital of St. Antonio. The lips were vermillioned; the countenance sunk; the complexion tarnished; the sleep interrupted by frightful dreams; no head-ache; mouth bitter, with ardent thirst, and red but moist tongue; pressure on the epigastrium, right hypochondrium, and anterior part of the left hypochondriac region caused exquisite pain. Respiration short, quick, and performed solely by the diaphragm; violent palpitation of the heart, with intermissions of its action, during which times the pupils would become fixed and dilated, the lips and face livid. The pulse was very unequal, intermittent, and sometimes hard.

5th day. The disease aggravated in all its symptoms, with the addition of cough and bloody sputa. General and local bleeding, without benefit.

On the 12th night the patient was delirious; and on the 20th, death put an end to her sufferings.

*Dissection.* The anterior portions of both lungs were condensed; in the cavity of the pericardium, a few ounces of limpid serum; but the membrane itself perfectly sound. The heart was double its natural size, soft, and flaccid. The left ventricle, in particular, was greatly dilated in volume, with extenuated parietes; the mitral valve was thickend and cartilaginous; the auriculo-ventricular aperture was contracted, and not more than six lines in diameter. On the posterior and superior surface of the left auricle were seen through the pericardium, which was free from disease, three or four small spheroidal tumours, in the muscular tissue, from two to three lines in diameter, and elevating the pericardial covering. On being cut into, there issued thick purulent matter streaked with blood. The auricular appendix of this side was dense and increased in volume, containing a sanious pus. The rest of the body presented nothing remarkable excepting some metacarpal and metatarsal phalanges.

We think there can be little doubt of this disease having been occasioned by a translation of arthritic or rheumatic irritation to the central organ of the circulation. We have lately seen a well marked case of this kind, which will be related in another place.

